# Recurrent episodes of atrioventricular nodal reentrant tachycardia: Sites of ablation success, ablation endpoint, and primary culprits for recurrence

**DOI:** 10.1002/joa3.13060

**Published:** 2024-05-14

**Authors:** Shu Hirata, Koichi Nagashima, Yoshiaki Kaneko, Shuntaro Tamura, Hitoshi Mori, Suguru Nishiuchi, Michifumi Tokuda, Tetsuma Kawaji, Tatsuya Hayashi, Takuro Nishimura, Masato Fukunaga, Jun Kishihara, Hidehira Fukaya, Jin Teranishi, Mitsuru Takami, Masato Okada, Naoko Miyazaki, Ryuta Watanabe, Yuji Wakamatsu, Yasuo Okumura

**Affiliations:** ^1^ Division of Cardiology, Department of Medicine Nihon University School of Medicine Tokyo Japan; ^2^ Department of Cardiovascular Medicine Gunma University Graduate School of Medicine Maebashi Japan; ^3^ Department of Cardiology Saitama Medical University International Medical Center Hidaka Japan; ^4^ Department of Cardiology Tenri Hospital Tenri Japan; ^5^ Department of Cardiology The Jikei University School of Medicine Tokyo Japan; ^6^ Department of Cardiology Mitsubishi Kyoto Hospital Kyoto Japan; ^7^ Division of Cardiovascular Medicine, Saitama Medical Center Jichi Medical University Shimotsuke Japan; ^8^ Department of Cardiovascular Medicine Tokyo Medical and Dental University Tokyo Japan; ^9^ Department of Cardiology Kokura Memorial Hospital Kitakyushu Japan; ^10^ Department of Cardiovascular Medicine Kitasato University School of Medicine Sagamihara Japan; ^11^ Department of Cardiovascular Medicine Japanese Red Cross Society Himeji Hospital Himeji Japan; ^12^ Division of Cardiovascular Medicine Kobe University Graduate School of Medicine Kobe Japan; ^13^ Cardiovascular Center Sakurabashi Watanabe Hospital Osaka Japan

**Keywords:** atrioventricular nodal reentrant tachycardia, leftward inferior extension, rightward inferior extension, slow pathway

## Abstract

**Background:**

Atrioventricular nodal reentrant tachycardia (AVNRT) sometimes recurs even after anatomical slow pathway (SP) ablation targeting the rightward inferior extension (RIE). This multicenter study aimed to determine the reasons for AVNRT recurrence.

**Methods and Results:**

Forty‐six patients were treated successfully for recurrent AVNRT. Initial treatment was for 38 slow‐fast AVNRTs, 3 fast‐slow AVNRTs, 2 slow‐slow AVNRTs, 2 slow‐fast and fast‐slow AVNRTs, and 1 noninducible AVNRT. All initial treatments were of RF application to the RIE; SP elimination was achieved in 11, dual AVN physiology was seen in 29, and AVNRT remained inducible in 5. The recurrent AVNRTs included 34 slow‐fast AVNRTs, 6 fast‐slow AVNRTs, 3 slow‐slow AVNRTs, 2 slow‐fast and fast‐slow AVNRTs, and 1 slow‐fast and slow‐slow AVNRTs. Successful ablation site was within the RIE in 39 and left inferior extension in 7. In 30 of 39, the successful RIE site was in the same area or higher than that of the initial procedure.

**Conclusion:**

For a high majority (around 85%) of patients in whom AVNRT recurs after initial ablation success, the site of a second successful procedure will be within the RIE even though the RIE was originally targeted. Furthermore, a high majority (around 86%) of sites of successful ablation will be higher than those originally targeted.

## Graphical abstract

Eighty‐five percent of a successful re‐ablation site will remain within the rightward inferior extension (RIE). Furthermore, 86% of successful RIE sites will be higher than those initially targeted.

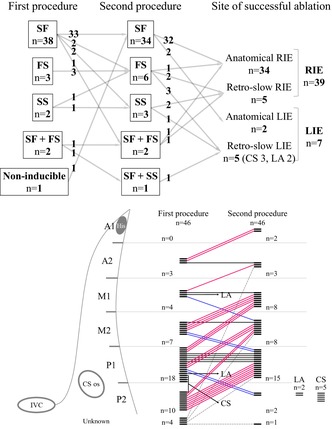

## 1 INTRODUCTION

Anatomical slow pathway (SP) ablation is a well‐accepted strategy for the treatment of atrioventricular nodal reentrant tachycardia (AVNRT).^1^, ^2^ Targeting the rightward inferior extension (RIE) of the SP offers a success rate of 96% to 98%,^3^, ^4^, ^5^ but AVNRT recurs in some cases even after a successful ablation procedure. Such recurrence may be due to the fact that endpoints of the SP ablation procedure remain controversial; junctional rhythm during RF energy application and absence of dual AV nodal physiology at the end of the procedure are not specific markers of ablation success.^2^ Also, anatomical variants of the SP, such as leftward inferior extension (LIE), a superior SP, and an inferolateral left atrial SP, have been reported in some (<5%) of AVNRT cases in recent years. These may contribute to the recurrence of AVNRT.^3^, ^4^, ^5^ Despite previous investigations into factors contributing to the recurrence of AVNRT, the optimal ablation site and endpoint of the second procedure in patients who experience recurrence after initial ablation success have not been fully elucidated. Therefore, we conducted a retrospective multicenter study to determine reasons for AVNRT recurrence by comparing the forms of AVNRT, the sites of successful ablation, and the ablation endpoints between the first and second procedures.

## 2 METHODS

### 2.1 Study patients

Forty‐six patients who, between January 2010 and December 2022, underwent a second procedure for AVNRT that recurred 363 (50, 991) days after an initial ablation procedure were identified from the medical records of 13 clinical centers. Among these 46 patients, 40 underwent the initial procedure at the same hospital. Additionally, during this period, a total of 3663 patients across the 13 clinical centers received their initial AVNRT ablation. Based on these treatment data, the recurrence rate of AVNRT is suggested to be 1.1%. The second procedure was successful in terms of modifying the SP in all 46 patients, and none suffered recurrence during the 6 month follow‐up period. Data collection and analysis were approved by the ethics committee of Nihon University Itabashi Hospital and that of each participating institution. Patients included had approved the use of their data for research purposes through an opt‐out method.

### 2.2 Electrophysiologic study

Electrophysiologic studies were performed under conscious sedation: A 4‐ to 10‐pole electrode catheter was inserted through the right femoral vein into the high right atrium and right ventricular (RV) apex and to the His bundle, and a 10‐ or 20‐pole catheter was inserted through the right jugular vein into the coronary sinus (CS). Bipolar intracardiac potentials were digitally recorded (LabSystem PRO, Bard Electrophysiology, Lowell, MA, or CardioLab EP, GE Healthcare, Wilmington, MA) at a paper speed of 100–200 mm/s through a bandpass filter of 30–500 Hz. Bipolar pacing was performed at a current of 10 mA and pulse width of 2 ms.

After exclusion of atrial tachycardia, by observance of a V‐A‐V response after RV overdrive pacing during tachycardia^6^ or of so‐called ventriculoatrial (VA) linking after differential atrial overdrive pacing,^7^ AVNRT was diagnosed if none of the following was present: (1) advancement or delay of the His timing of >10 ms or termination of the tachycardia on delivery of a scanned single premature ventricular contraction (PVC) during His‐bundle refractoriness; (2) an uncorrected/corrected postpacing interval (PPI) minus a tachycardia cycle length (TCL) of <115 ms/<110 ms;^8^, ^9^ and (3) orthodromic His or septal ventricular capture during RV overdrive pacing.^10^ Entrainment of the tachycardia was attempted by RV overdrive pacing at a cycle length of 10–30 ms less than the TCL.

Patients' AVNRTs were categorized according to their form as slow‐fast AVNRT (His‐atrial [HA] interval ≤70 ms), fast‐slow AVNRT (HA interval >70 ms, atrio‐His, AH/HA ratio <1, and AH interval <200 ms), or slow‐slow AVNRT (HA interval >70 ms, AH/HA ratio >1, and AH interval >200 ms).^11^

### 2.3 Ablation procedure

**FIGURE 1:**
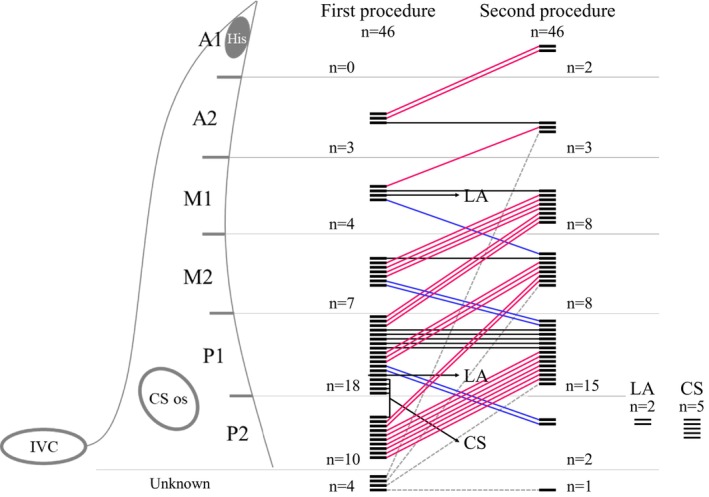
(Left) Division of the anatomical area of the slow pathway (area from the lower margin of the CS ostium to the His bundle recording site) into 6 level‐based segments: A1, A2, M1, M2, P1, and P2. The site of successful ablation was assigned to the pertinent segment. (Right) Line graph plotting anatomical levels at which the slow pathway was successfully ablated initially and then during the second procedure. Pink‐colored lines indicate that the site of ablation success was higher in the second procedure than in the first procedure, and blue‐colored lines indicates the opposite. Black‐colored lines indicate that site of ablation success was at the same level in both the initial and second procedure. CS, coronary sinus; IVC, inferior vena cava; LA, left atrium.

For SP modification, the so‐called integrated approach was used, combining anatomical landmarks and electrographic recordings.^12^ Radiofrequency (RF) energy (*n* = 40) or cryothermal energy (*n* = 6) was applied by conventional approach when an atrial/ventricular electrogram ratio of <0.5 was obtained during sinus rhythm with a 3.5–4.5 mm tip RF catheter or 7Fr 6 mm tip cryoablation catheter (Freezor Xtra, Medtronic, Minneapolis, MN).^13^, ^14^, ^15^ RF power was titrated from 25 to 40 W with maximum temperature of 60°C, and RF delivery duration was set for up to 60 s until junctional rhythm was elicited. Cryothermal energy at a catheter tip temperature of −80°C was applied for 240 s at the site where cryomapping at −30°C terminated the AVNRT or eliminated the dual AVN physiology. After thawing of the targeted lesion to body temperature, additional 240 s cryoenergy applications were performed at the same site. If AV block occurred, the RF energy/cryoenergy delivery was immediately stopped. After RF/cryoenergy application, the dual AV nodal physiology and AVNRT inducibility were reassessed with/without isoproterenol infusion. RF/cryoenergy delivery was gradually extended superiorly if AVNRT remained inducible. If AVNRT remained inducible despite extensive RF application to the RIE, a within the CS or left atrial approach was attempted at the operator's discretion. Acute success was defined as complete SP elimination or an AH jump with/without a single AVN echo. The site of successful ablation was defined as the site of RF energy/cryoenergy delivery where the AVNRT was rendered noninducible or the final ablation site. The area from the lower margin of the CS ostium to the His bundle recording site was divided anatomically into 6 level‐based segments: A1, A2, M1, M2, P1, and P2 (Figure 1, left panel). The site of successful ablation was assigned to 1 of the 6 segments.

### 2.4 Statistical analysis

Study data are shown as the numbers (and percentages) of patients or as mean ± SD or median (25th, 75th percentile) values. Differences in variables between the initial procedure and second procedure were analyzed by paired Student *t*‐test. All statistical analyses were performed with JMP 15 software (SAS Institute, Cary, NC).

## 3 RESULTS

### 3.1 Initial procedure

**FIGURE 2:**
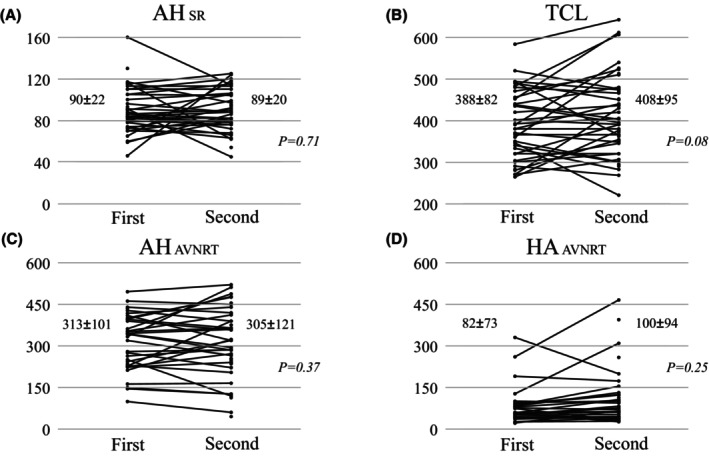
(A) Atrio‐His (AH) interval during sinus rhythm, (B) tachycardia cycle length (TCL), and (C) AH and (D) His‐atrial intervals during atrioventricular nodal reentrant tachycardia at the time of the initial and second procedures.

**FIGURE 3:**
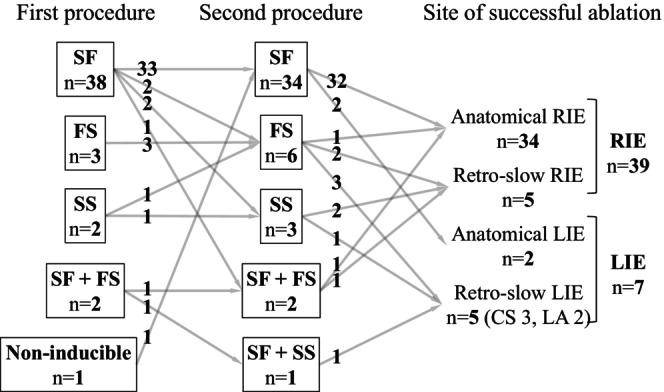
The forms of atrioventricular nodal reentrant tachycardia at first and second procedures and sites of successful ablation of the recurrent atrioventricular nodal reentrant tachycardia at second procedure. FS, fast‐slow; LIE, left inferior extension; RIE, right inferior extension; SF, slow‐fast; SS, slow‐slow.

**FIGURE 4:**
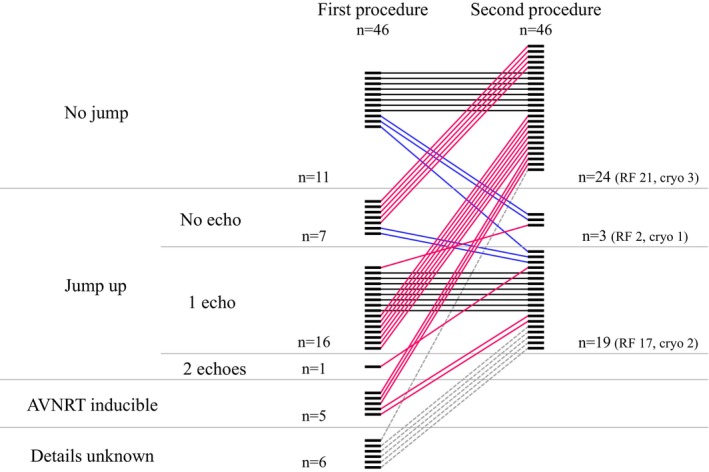
Postprocedural endpoints of the initial and second ablation procedures. Pink‐colored lines indicate patients for whom the ablation endpoint improved, and blue‐colored lines indicate the opposite. Black‐colored lines indicate patients for whom the endpoint did not change. Dotted lines indicate patients for whom the endpoint of the initial procedure is unknown.

Patient characteristics, electrophysiologic features of the AVNRT, and details pertaining to the initial ablation procedure are shown in Table 1 and Figures 2 and 3. At the time of the initial procedure, the mean AH interval during sinus rhythm was 90 ± 22 ms (Figure 2A). AVNRT with a mean TCL of 388 ± 82 ms (Figure 2B) induced at that time and was of the slow‐fast form in 38 (82%) patients, fast‐slow form in 3 (7%), slow‐slow form in 2 (4%), and both the slow‐fast form and fast‐slow form in 2 (4%) (Figure 3). The AVNRT was not inducible during the initial procedure in 1 (2%) patient, although a very short RP tachycardia had been clinically documented. RF ablation was performed in all 46 patients. In 1 patient with fast‐slow AVNRT and 1 patient with both slow‐fast AVNRT and fast‐slow AVNRT, RF energy was applied to the site of earliest atrial activation during the tachycardia. Junctional rhythm during RF ablation was observed in 38 of 45 patients (89%), except for one case with unknown details. As shown in Figure 4 (left panel), complete SP elimination characterized by absence of an AH jump was achieved in 11 (24%) patients, and dual AVN physiology was seen in 29 (63%) (AH jump without an echo beat in 7, with a single echo beat in 16, with 2 echo beats in 1, and with AVNRT remaining inducible in 5 patients). Postablation details were not available for 6 (13%) patients.

**TABLE 1:** Patient characteristics, electrophysiologic findings, and details pertaining to the initial ablation procedure (*n* = 46 patients).

Age (years)	53 ± 18
Male gender	21 (46%)
AH interval (ms) during sinus rhythm	90 ± 22
Electrophysiologic features of the AVNRT	
TCL (ms)	388 ± 82
Slow‐fast form	38 (83%)
Fast‐slow form	3 (7%)
Slow‐slow form	2 (4%)
Slow‐fast and fast‐slow forms	2 (4%)
Noninducible	1 (2%)
Ablation target	
RIE area (anatomical approach)	44 (96%)
Earliest atrial activation site in the RIE	2 (4%)
Number of RF or cryoenergy applications to success	8 (5, 14)
Postablation findings	
No AH jump	11 (24%)
AH jump without an AVN echo beat	7 (15%)
AH jump with a single AVN echo beat	16 (35%)
AH jump with 2 AVN echo beats	1 (2%)
AVNRT inducible	5 (11%)
Unknown	6 (13%)

*Note*: Values are mean ± SD, median (25th, 75th percentiles), or *n* (%).

Abbreviations: AH, atrio‐His; AVNRT, atrioventricular nodal reentrant tachycardia; RF, radiofrequency; RIE, right inferior extension; TCL, tachycardia cycle length.

### 3.2 Features of the recurrent AVNRTs

Electrophysiologic features of the recurrent AVNRTs and details pertaining to the recurrent AVNRT and second ablation procedure are shown in Table 2 and Figure 1 (right panel) through Figure 4. The AH interval during sinus rhythm did not differ from that at the time of the initial procedure (Figure 2A). As shown in Table 2, induced AVNRTs were of the slow‐fast form in 34 (74%) patients, fast‐slow form in 6 (13%), slow‐slow form in 3 (7%), both the slow‐fast and fast‐slow forms in 2 (4%), and both the slow‐fast and slow‐slow forms in 1 (2%). Slow‐fast recurrent AVNRT was diagnosed in 33 of the 38 patients initially diagnosed with slow‐fast AVNRT and in the 1 patient in whom the AVNRT had been noninducible. In 5 of the 38 patients initially diagnosed with slow‐fast AVNRT, the recurrent AVNRT was of the fast‐slow form in 2, slow‐slow form in 2, and both slow‐fast and fast‐slow forms in 1. All 3 patients who had been diagnosed with fast‐slow AVNRT were again diagnosed with fast‐slow AVNRT. Of the 2 patients who had been diagnosed with slow‐slow AVNRT, 1 was diagnosed with the slow‐slow form and the other with the fast‐slow form. Of the 2 patients in whom both the slow‐fast and fast‐slow forms were inducible during the first procedure, 1 exhibited both the slow‐fast and fast‐slow forms and 1 exhibited both the slow‐fast and slow‐slow forms (Figure 3). Neither the AH interval during sinus rhythm, TCL, AH interval during AVNRT, nor HA interval during AVNRT differed in comparison to that at the time of the initial procedure (Figure 2A–D).

**TABLE 2:** Electrophysiologic findings and details pertaining to the second ablation procedure (*n* = 46 patients).

Electrophysiologic findings	
AH interval (ms) during sinus rhythm	89 ± 20
HV interval (ms) during sinus rhythm	48 ± 9
Dual AV nodal physiology	
Antegrade	37 (80%)
Retrograde	7 (15%)
AVNRT features	
TCL (ms)	408 ± 95
Slow‐fast form	34 (74%)
Fast‐slow form	6 (13%)
Slow‐slow form	3 (7%)
Slow‐fast and fast‐slow forms	2 (4%)
Slow‐fast and slow‐slow forms	1 (2%)
Site of successful ablation	
RIE	39 (85%)
Anatomical approach	34 (74%)
Earliest atrial activation site	5 (11%)
LIE	7 (15%)
Anatomical approach	2 (4%)
Earliest atrial activation site	5 (11%)
Number of RF energy or cryoenergy applications to success	7 (4, 15)
Postablation findings	
AH interval (ms)	87 ± 19
No AH jump	24 (52%)
AH jump without an AVN echo beat	3 (7%)
AH jump with a single AVN echo beat	19 (41%)

*Note*: Values are mean ± SD, median (25th, 75th percentiles), or *n* (%). Red color signifies the AH interval during sinus rhythm for postablation, It means that there is no significant change compared to preablation.

Abbreviations: AH, atrio‐His; AV, atrioventricular; AVNRT, atrioventricular nodal reentrant tachycardia; HV, His‐ventricular; LIE, left inferior extension; RF, radiofrequency; RIE, right inferior extension; TCL, tachycardia cycle length.

### 3.3 Details of the second procedure

RF ablation and cryoablation were performed for the recurrent AVNRT in 40 patients and 6 patients, respectively. Approaches to the second procedure are shown in Figure 3. Anatomical Rcon was attempted in 32 patients with slow‐fast AVNRT, 1 with fast‐slow AVNRT, and 1 with both slow‐fast AVNRT and fast‐slow AVNRT. Anatomical LIE ablation was performed in 2 patients with slow‐fast AVNRT. The electrophysiologic approach targeting the earliest atrial activation site during AVNRT or RV pacing was performed in the RIE region in 2 patients with fast‐slow AVNRT, 2 patients with slow‐slow AVNRT, and 1 patient with both slow‐fast and fast‐slow AVNRT, and in the LIE region (within the CS in 3, in the left atrium in 2) in 3 patients with fast‐slow AVNRT, 1 with slow‐slow AVNRT, and 1 with both slow‐fast and slow‐slow AVNRT. In 39 patients who underwent successful RIE ablation, the average number of ablation points was 8 ± 6. For 7 patients achieving successful ablation in the LIE region, and RF application was administered inside the CS (*n* = 5) and LA (*n* = 2), following an initial attempt of 11 ± 5 RF applications in the RIE area. Notably, no patient received RF applications in both the CS and LA.

The areas of successful ablation of the recurrent AVNRT are shown in Figure 1. Among the 39 patients who underwent successful ablation in the RIE region, 86% (30 out of 35) had their ablation site either in the identical area (*n* = 8) or higher (anterior) compared to the initial procedure (*n* = 22), excluding four patients with unknown initial successful ablation sites. The remaining 5 patients had their successful ablation sites lower (posterior) than the initial site. Among the 7 patients for whom successful ablation was in the LIE region, the site was within the CS in 5 (2 with slow‐fast AVNRT, 2 with fast‐slow form AVNRT, and 1 with both slow‐fast and slow‐slow AVNRT) and in the left atrium in 2 (1 with slow‐slow AVNRT and 1 with fast‐slow AVNRT). Junctional rhythm was observed during RF application in 35 of 38 patients (92%); however, in 3 patients, the assessment was indeterminate due to RF application during atrial pacing or tachycardia. In contrast, junctional rhythm was not observed during cryoablation in any of the 5 patients. The AH interval after the procedure was 87 ± 19 ms. In 5 patients (11%), the AH interval was prolonged by more than 10 ms compared to before the ablation. For all these 5 patients, the successful ablation site was located higher than in the initial procedure.

Ablation endpoints are shown in Figure 4. Acute success was achieved during the second ablation procedure in all cases. By the end of the second procedure, complete SP elimination characterized by absence of an AH jump was achieved in 24 (52%) patients (RF ablation = 21, cryoablation = 3), and dual AVN physiology was seen in 22 (48%) with no echo beat in 3 (RF ablation = 2, cryoablation = 1) and a single echo beat in 19 (RF ablation = 17, cryoablation = 2). More than 1 echo beat was not seen in any patient. In 15 of the 29 (52%) patients with an AH jump with/without an echo beat at the end of the initial procedure, no AH jump was observed. In contrast, the 8 (73%) patients without AH jump and 8 (50%) patients with AH jump and 1 echo beat, the same endpoint was achieved. In all cases, noninducibility of AVNRT with isoproterenol administration was confirmed. No serious complication occurred. In all cases, no further recurrence of AVNRT was observed during the follow‐up period of 256 (39, 1034) days.

## 4 DISCUSSION

The main findings of this study were as follows: (1) The recurrent AVNRT was of the same form as that treated initially in 84% of patients; (2) the site of successful ablation for the recurrent AVNRT was in the RIE area in 85% of patients even though the RIE area was targeted in the initial procedure; (3) LIE area ablation within the CS or the left atrium was required in 15% of patients for whom the AVNRT recurred; (4) the site of successful ablation in the RIE was at the same level or higher than that in the initial procedure in 86% of patients; and (5) in 52% of patients in whom AH jump with/without any echo was seen at the end of the initial procedure, no such jump was observed at the end of the second procedure.

In the current clinical context, with a high success rate of 96%–98% for AVNRT ablation, electrophysiologists have begun to consider anatomical variants when a second procedure is scheduled for recurrent AVNRT.^3^, ^4^, ^5^ These anatomical variants may pose challenges during the ablation procedure, and awareness of their presence can aid in achieving success. In atypical forms of AVNRT, such as the fast‐slow and slow‐slow forms, the location of the SP as a retrograde limb of the AVNRT can be easily mapped as the earliest atrial activation site, even when an anatomical variant of the SP is present. However, in the majority of cases of AVNRT, that is, in cases of slow‐fast AVNRT, mapping of the SP as an anterograde limb of the AVNRT can be challenging because it cannot be reliably delineated through simple activation mapping. More advanced mapping techniques, such as incorporation of atrial extrastimulus or atrial entrainment pacing, may be necessary to identify the location of the SP in these cases. However, these mapping techniques require careful real‐time interpretation to assess the tiny interval of the atrial reset or account for prolongation of the postpacing interval due to the decremental conduction over the AVN. Therefore, our finding that the site of successful ablation of the recurrent AVNRT was within the RIE region in 85% of our patients, despite the fact that the prior site of success was in the same region, provides electrophysiologists with valuable insight. Furthermore, 86% of sites of successful ablation in the RIE area during the second procedure were at the same or a higher level than sites of successful ablation during the initial procedure. It might be that the previous RF energy applications were insufficient for fear of AV block. The similarity in AH and HA intervals associated with the initial procedure and those associated with the second procedure suggest that the initial SP modification was less than curative, supporting our hypothesis. In 5 of the 22 patients, however, successful ablation at a site higher than in the initial procedure led to an AH prolongation of more than 10 ms. This indicates that while targeting a higher area may be effective for the majority of these patients, it could also raise the risk of AV block. In these cases, cryoablation may be an option for recurrent slow‐fast AVNRT because cryoablation is of similar efficacy and poses a relatively low risk of AV block.^16^ Notably, the site of successful ablation in the RIE was lower than the site of previous success in the remaining 14% of patients. It might be necessary to apply RF energy initially to the lower part of the triangle of Koch and then gradually extend application to the higher area even during the second treatment. Moreover, if the AVNRT is still inducible after considerable RF applications in the RIE, LIE ablation within the CS should be considered. In contrast, all recurrent atypical AVNRTs were also atypical AVNRTs when treated initially. In 42% of these cases, the site of ablation success was in the LIE. Thus, the major reason for ablation failure in cases of atypical AVNRT might be a change in the retrograde SP to another SP variant rather than insufficient RF applications in the RIE region. The earliest atrial activation site should be targeted as in the initial ablation procedure.

None of our patients showed more than 1 AVN echo beat after the second procedure. Additionally, in 52% of patients in whom AH jump with/without any echo was observed at the end of the initial procedure, no jump was seen at the end of the second procedure. This finding aligns with previous reports, suggesting that an endpoint characterized by an AH jump along with a single echo beat does not invariably indicate a successful ablation.^2^ One of the largest multicenter studies of SP ablation for AVNRT showed noninducibility of AVNRT with isoproterenol administration at the end of the procedure, rather than induction of junctional rhythm during RF energy application or absence of dual AVN physiology to be the most reliable marker of ablation succes.^2^ It might be that the endpoint for successful ablation differs from patient to patient. The appropriate endpoint should be identified and achieved on a case‐by‐case basis.

### 4.1 Limitations

Our findings should be interpreted in light of our study limitations. The first is its execution as a small, retrospective investigation. However, we believe our findings are clinically meaningful because the sample size of 46 extrapolates to a population of 2300 patients treated by ablation for AVNRT and success rate of 98%. Our findings are based on meticulous analysis of the electrophysiologic characteristics of the arrhythmias and the electrocardiographic measurements. Second, it is conceivable that a similar analysis involving a very large patient group would yield different diagnostic cut‐off values, but we believe this to be unlikely.

## 5 CONCLUSIONS

For a high majority (around 85%) of patients in whom AVNRT recurs after initial ablation success, the site of a second successful ablation procedure will be in the RIE area even though the RIE area was targeted during the initial procedure. Furthermore, a high majority (around 86%) of sites of successful ablation will be higher than those targeted in the initial procedure. LIE area ablation within the CS or the left atrium will most likely be required in the remaining (approximately15%) patients.

## CONFLICT OF INTEREST STATEMENT

Authors declare no conflict of interests for this article.

## ETHICS STATEMENT

Approval of research protocol: The study was approved by the Institutional Review Board of Nihon University Itabashi Hospital, approval number: RK‐221108‐7.

## PATIENT CONSENT STATEMENT

An opt‐out system was used to obtain the patients' content for the use of their clinical data for research purposes.

## CLINICAL TRIAL REGISTRATION

N/A.
